# *RAS/RAF* mutations and microsatellite instability status in primary colorectal cancers according to *HER2* amplification

**DOI:** 10.1038/s41598-024-62096-x

**Published:** 2024-05-19

**Authors:** Sun Mi Lee, Hyunjoo Oh

**Affiliations:** 1 Department of Pathology and Laboratory Medicine, Indiana University School of Medicine, 350 W. 11th Street, Indianapolis, IN 46202 USA; 2 Department of Pathology, Jeju National University Hospital, Jeju-si, South Korea; 3 Department of Internal Medicine, Jeju National University Hospital, Jeju-si, South Korea

**Keywords:** Gastroenterology, Oncology

## Abstract

*HER2* amplification-associated molecular alterations and clinicopathologic features in colorectal cancers (CRCs) have not been well established. In this study, we assessed the prevalence of *HER2* amplification and microsatellite instability (MSI) status of 992 patients with primary CRC. In addition, molecular alterations of *HER2* amplified and unamplified CRCs were examined and compared by next-generation sequencing. *HER2* amplifications were found in 41 (4.1%) of 992 primary CRCs. *HER2* amplification was identified in 1.0% of the right colonic tumors, 5.1% of the left colonic tumors, and 4.8% of the rectal tumors. Approximately 95% of *HER2* amplification was observed in the left colon and rectum. Seven (87.5%) of eight metastatic tumors showed *HER2* amplification. Most clinicopathologic features were unrelated to *HER2* amplification except tumor size and MSI status. All 41 *HER2* amplified CRCs were microsatellite stable. In a molecular analysis of frequently identified somatic mutations in CRCs, *HER2* amplified CRCs showed a lower rate of *KRAS* mutations (24.4%) but a higher rate of *TP53* mutations (83%) than unamplified CRCs. No *BRAF* and *NRAS* mutations were identified in *HER2* amplified CRCs. Our study suggests that *HER2* amplified CRCs are mutually exclusive of MSI and harbor less frequent *KRAS/NRAS/BRAF* mutations but frequent *T53* mutations.

## Introduction

The frequency of *HER2* overexpression/amplification ranges from 1.3 to 6.3% of unselected colorectal cancers (CRCs)^1–3^. *HER2* amplification leading to *HER2* protein overexpression is associated with downstream activated signaling pathways, leading to dysregulated cell proliferation, including breast cancer, esophagogastric cancer, and CRC^4^. Recently, the U.S. Food and Drug Administration approved tucatinib + trastuzumab combination therapy for patients with *HER2* amplified CRC^5^. Although the prevalence of *HER2* amplification in CRCs is relatively lower than in other cancers, detecting *HER2* amplification in CRCs becomes inevitable for selecting patients who may benefit from *HER2*-targeted therapy.

Microsatellite instability (MSI) is a hypermutable phenotype caused by mismatch repair deficiency, which accounts for 10–15% of all CRCs^6^. The MSI phenotype involves the primary loss of function of proteins that usually repair DNA base-pair mismatches, mainly MLH1, MSH2, MSH6, and PMS2. In association with other molecular alterations identified in CRCs, the microsatellite instability-high (MSI-H) phenotype has shown to be significantly associated with *BRAF* V600E mutations but tends to correlate inversely with *KRAS* mutation^7^. In contrast, the possible correlation between *HER2* amplification and MSI status has not been well investigated. A few molecular studies of CRCs and gastric cancers suggest that most *HER2* amplified tumors were microsatellite stable (MSS), representing a negative correlation between *HER2* amplification and MSI in gastrointestinal cancers^8,9^.

According to the National Comprehensive Cancer Network (NCCN) guideline, all patients with metastatic CRC who are eligible for anti-epidermal growth factor receptor (EGFR) inhibitors should have the tumor tissue tested for *BRAF* and *RAS* mutations^10^. Because *BRAF* and *RAS* mutations, including *KRAS* and *NRAS*, are associated with primary resistance to anti-EGFR therapy. The current guideline recommends that only patients with *RAS* wild type should be treated with anti-EGFR inhibitors after evaluation of *RAS* mutations. Therefore, evaluation of *RAS* mutations in *HER2* amplified CRCs can provide further information to select therapeutic options, including *HER2*-targeted therapy.

The correlations between *HER2* status and different clinicopathologic features have been controversial. Some studies suggest that *HER2* overexpression is associated with aggressive biological behavior, which includes profound invasion, lymphatic invasion, lymph node metastasis, distant metastasis, and distal colon location^3,11,12^. In contrast, other studies demonstrated no correlations between clinicopathologic features, including tumor location and *HER2* overexpression/amplification^13–15^. Thus, we investigate to find any correlations between *HER2* amplification and clinicopathologic features in a cohort study of 992 patients with primary CRCs.

The main purpose of this study was to better elucidate the prevalence of *HER2* expression and amplification and associated clinicopathologic features in patients with primary unselected CRC. After the selection of *HER2* amplified CRC cases, microsatellite instability status and molecular alterations of *HER2* amplified and unamplified CRCs after matching tumor sites were compared by next-generation sequencing.

## Results

### Expression and amplification of *HER2*

**Figure 1:**
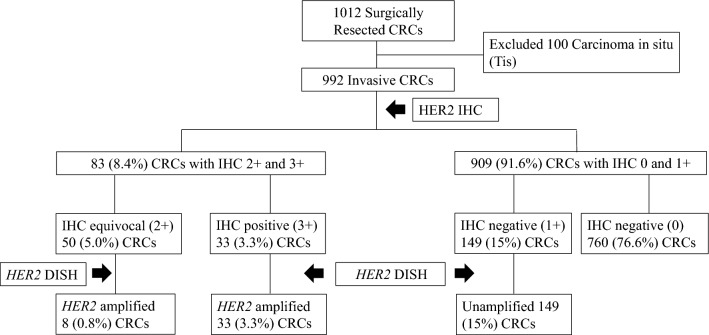
A flow chart showing each step of detecting *HER2* expressed and amplified tumors in 992 invasive CRCs using immunohistochemistry and DISH. Among 992 unselected CRCs, there were 149 (15%) tumors with 1 + labeling, 50 (5%) tumors with 2 + labeling, and 33 (3.3%) tumors with 3 + labeling by immunohistochemistry. *HER2* DISH was performed on tumors with all 1 + , 2 + , and 3 + labeling. Finally, 41(4.1%) tumors were found to be *HER2* amplified.

**Figure 2:**
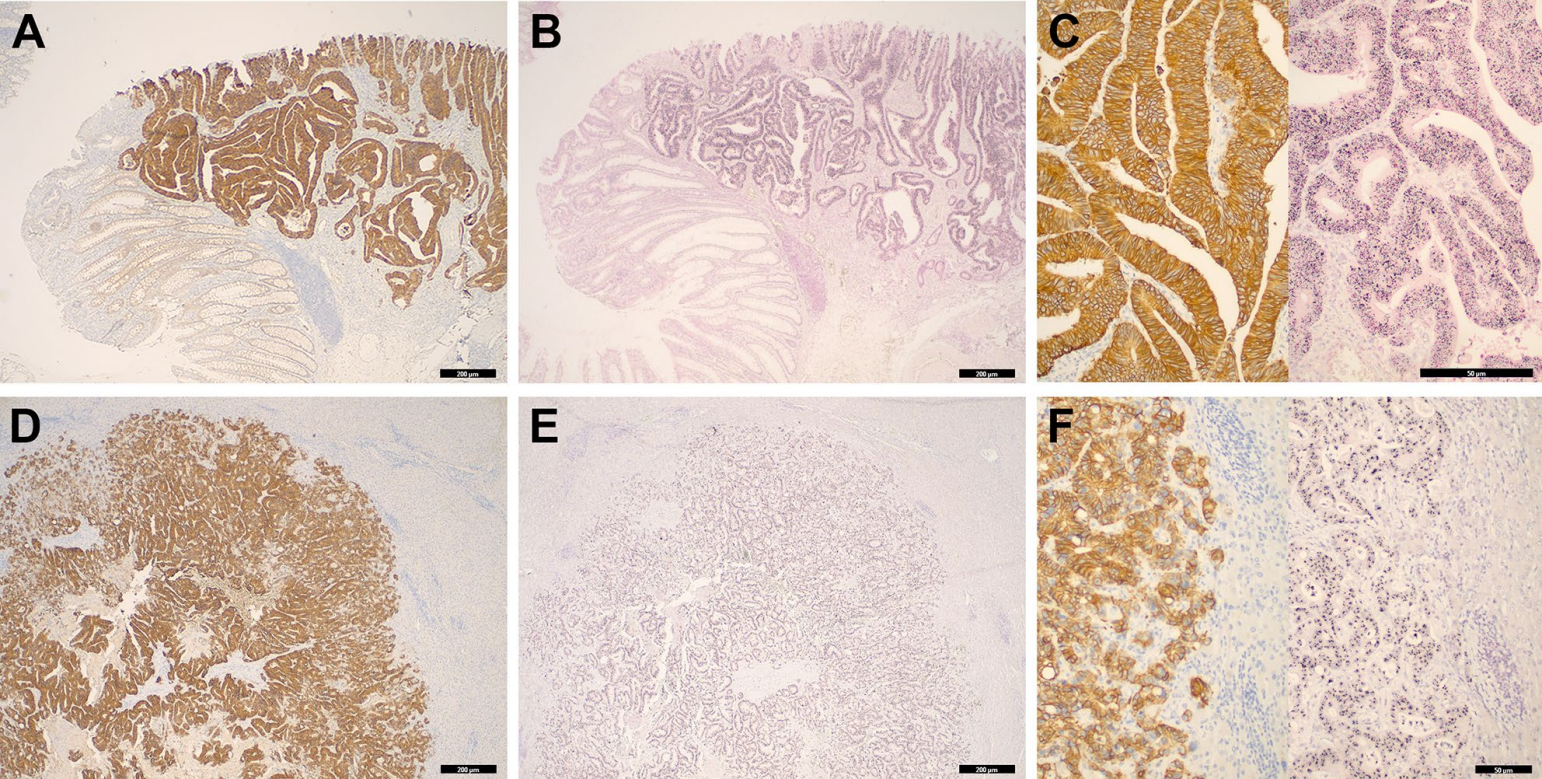
Representative micrographs of *HER2* immunohistochemistry and DISH on the primary and metastatic CRCs are shown. (**A**) The primary CRC reveals a diffuse and strong staining pattern (3 +) of *HER2* immunostaining at low magnification (4×). (**B**) The corresponding primary tumor shows diffusely located individuals and clusters of black dots within neoplastic cells on DISH. (**C**) At high magnification (40×), neoplastic cells exhibit intense membranous staining (3 +) of *HER2* (left) and highly clustered black dots (right) representing amplified *HER2* gene signals. (**D**) Seven of eight metastatic CRCs harbor *HER2* amplification on DISH, representing 87.5% of the concordance rate between the primary and metastatic CRCs. The metastatic CRC in the liver displays a diffuse and strong staining pattern (3 +) of *HER2* at low magnification (4×). (**E**) The corresponding metastatic tumor shows diffuse and strong black dots on DISH. (**F**) At high magnification (20×), neoplastic cells within the liver parenchyma reveal a strong membranous staining pattern (3 +) of *HER2* (left) and highly clustered black dots (right) representing amplified *HER2* gene signals.

A flow chart of *HER2* expression and amplification of 992 primary CRCs revealed by immunohistochemistry and dual-colored in situ hybridization (DISH) is illustrated in Fig. 1. Among 992 primary CRCs, there were 149 (15%) tumors with 1 + labeling, 50 (5.0%) tumors with 2 + labeling, and 33 (3.3%) tumors with 3 + labeling. DISH was performed on 232 tumors with 1 + , 2 + , and 3 + labeling. All 33 (3.3%) tumors with 3 + labeling revealed high *HER2* amplifications on DISH. Among 50 (5%) tumors with 2 + labeling, 8 (0.8%) tumors were found to have *HER2* amplifications on DISH. Among 41 (4.1%) *HER2* amplified tumors, there were 36 (3.6%) tumors with high amplifications and 5 (0.5%) tumors with low amplifications on DISH. During follow-up, eight patients underwent surgical resections of metastatic lesions. Additional immunohistochemistry and DISH were performed on eight metastatic tumors in the liver and lung. Seven (87.5%) of eight metastatic tumors showed *HER2* amplification on DISH. The concordance rate of *HER2* amplification between the primary and metastatic CRCs was 87.5%. Immunohistochemistry and DISH for *HER2* on primary and metastatic CRC tumors are described in Fig. 2.

### Clinicopathologic features and MSI status of *Her2* amplified and unamplified CRCs

**Figure 3:**
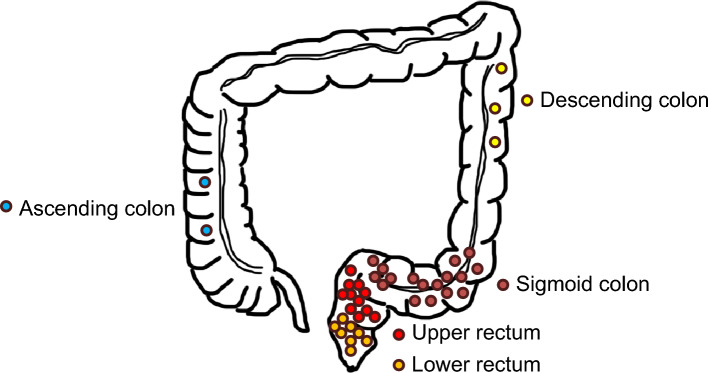
This illustration shows the anastomotic site where *HER2* amplified tumors occur in the colorectum. The most common primary tumor site was the left colon (48.8%), followed by the rectum (46.3%) and the right colon (4.9%). In *HER2* amplified left colonic tumors, seventeen (85%) were observed in the sigmoid colon. Moreover, in *HER2* amplified rectal tumors, eleven (57.9%) occurred in the upper rectum, a median of 11 cm (range, 7–15 cm) located above the anal verge. All two (4.9%) *HER2* amplified tumors were found in the ascending colon.

Among 992 patients with primary CRCs, forty-one (4.1%) patients had *HER2* amplified CRC. *HER2* amplifications were identified in 1.0% (2/193) of the right colonic tumors, 5.1% (20/394) of the left colonic tumors, and 4.8% (19/400) of the rectal tumors. In clinicopathologic features of 41 patients with *HER2* amplified CRC, the patients consisted of twenty-four (58.5%) men and seventeen (41.5%) women (male to female ratio: 1.4), with a mean age of 60 years. One patient had a history of ulcerative colitis. Four patients had a history of prior sigmoid colon cancer, lung adenocarcinoma, early gastric adenocarcinoma, and gastric gastrointestinal stromal tumor. Six (14.6%) patients with *HER2* amplified CRC received neoadjuvant chemoradiation before the surgery. The median tumor size was 3.5 cm (0.8–7.8 cm in greatest dimension). There was one case with two synchronous tumors in the cecum and ascending colon/right-sided colon. The most common primary tumor site was the left colon (48.8%), followed by the rectum (46.3%) and the right colon (4.9%). In twenty (48.8%) amplified left-sided tumors, seventeen (85%) were observed in the sigmoid colon. In nineteen (46.3%) amplified rectal tumors, eleven (57.9%) were observed in the upper rectum, a median of 11 cm (range, 7–15 cm) located above the anal verge; eight (42.1%) tumors were found in the lower rectum, a median of 3.2 cm (range, 2–5.9 cm) above the anal verge. Two (4.9%) *HER2* amplified tumors were observed in the ascending colon. The anastomotic sites of *HER2* amplified primary CRCs are illustrated in Fig. 3. Twenty-five (60.1%) *HER2* amplified tumors were in the T3 and T4 categories. Metastatic disease was noted in 4 (9.8%) patients at the time of diagnosis, with the liver being the most common organ involved. Fourteen (34.1%) patients with *HER2* amplified CRC were diagnosed with pathologic stages III and IV. In the comparison analysis of *HER2* amplified and unamplified CRCs, there were no statistical differences in most clinicopathologic features between the two tumor groups except tumor size and MSI status. All 41 *HER2* amplified CRCs were MSS phenotypes revealed by MSI testing compared to 100 (10.5%) MSI-H tumors in 951 unamplified CRCs. A comparison analysis of the clinicopathologic features of *HER2* amplified and unamplified tumor groups is detailed in Table 1.

**Table 1:** Comparison of clinicopathologic features between patients with *HER2* amplified versus unamplified CRC.

Clinicopathologic variables	*HER2* amplified 41 cases N (%)	*HER2* unamplified 951 cases N (%)	*p*-value
Sex			
Male	24 (58.5)	561 (59)	> 0.9999
Female	17 (41.5)	390 (41)	
Age, mean, year (range)	60 (34–82)	62 (19–94)	
Multifocality			
Single	40 (97.6)	912 (95.9)	> 0.9999
Multiple	1 (2.4)	39 (4.1)	
Tumor size, median, cm (range)	3.5 (0.8–7.8)	4.3 (0.2–18)	0.0319
Location			0.0684
Right	2 (4.9)	191 (20)	
Left	20 (48.8)	374 (39)	
Rectum	19 (46.3)	381 (40)	
Right and Left	0	5 (1)	
Differentiation			0.2122
WD	7 (17.1)	82 (8.6)	
MD	32 (78)	801 (84.2)	
PD	2 (4.9)	68 (7.2)	
T category			
T1	8 (19.5)	110 (11.6)	0.3208
T2	8 (19.5)	146 (15.4)	
T3	20 (48.8)	528 (55.5)	
T4	5 (12.2)	167 (17.5)	
Lymphovascular invasion			
Present	18 (43.9)	458 (48.2)	0.6345
Absent	23 (56.1)	493 (51.8)	
Perineural invasion			
Present	12 (29.3)	272 (28.6)	> 0.9999
Absent	29 (70.7)	679 (71.4)	
N category			
N0	28 (68.3)	527 (55.4)	0.1942
N1	10 (24.4)	273 (28.7)	
N2	3 (7.3)	151 (15.9)	
M category			
M0	37 (90.2)	856 (90)	> 0.9999
M1	4 (9.8)	95 (10)	
AJCC stage			
I	15 (36.6)	201 (21.1)	0.1072
II	12 (29.3)	304 (32)	
III	10 (24.4)	351 (36.9)	
IV	4 (9.8)	95 (10)	
MSI status			
MSS/MSI-L	41 (100)	851 (89.5)	< 0.0001
MSI-H	0	100 (10.5)	

WD, well differentiated; MD, moderately differentiated; PD, poorly differentiated; MSI, microsatellite instability; MSS, microsatellite stable; MSI-L, microsatellite instability-low; MSI-H, microsatellite instability-high.

### Molecular alterations between *HER2* amplified and unamplified CRCs

All forty-one primary CRCs with *HER2* amplification on DISH were examined using the 88-gene panel. Copy numbers of *HER2* ranged from 5 to 43 with a mean copy number of 13. There was no discordance between the results of DISH and next-generation sequencing in *HER2* amplification (100% concordance rate). No activating *BRAF* mutations were detected. Activating *KRAS* mutations were identified in 24.4% (10/41) amplified tumors, predominantly in codons 12 and 13, including p.G12D, p.G12V, and p.G13D. Other *RAS* genes, such as *NRAS* and *HRAS* mutations, were not detected. *TP53* mutations were identified in 82.9% (34/41) of amplified tumors without consistently mutated hot spots. *APC* mutations were found in 51.2% (21/41) of amplified tumors without mutated hot spots. *PIK3CA, ERBB2/3, SMAD4, PTEN*, and *FBXW7* mutations were detected in 17.1% (7/41), 7.3% (3/41), 4.9% (2/41), 2.4% (1/41), and 7.3% (3/41) of amplified tumors. Additionally, mutations in various genes, including *CDK4, ATM, KIT*, and so on, were occasionally detected*.* The majority of these mutations are of unknown functional consequences. Somatic mutations and gene alterations identified in 41 *HER2* amplified CRCs are listed in supplementary Table 1. 

**Figure 4:**
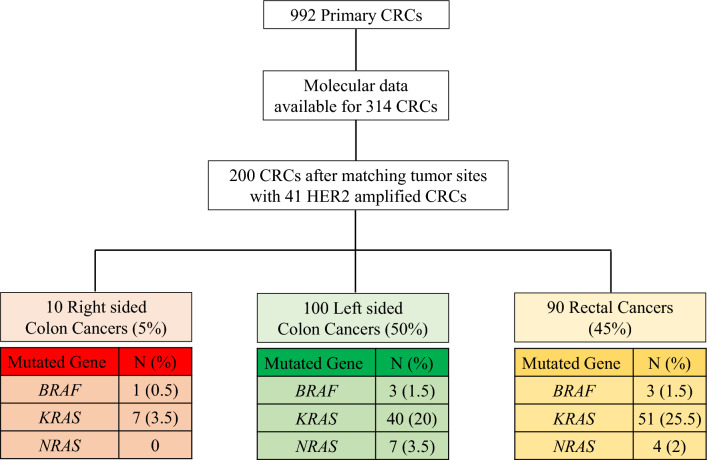
In order to compare molecular differences between *HER2* amplified and unamplified CRCs, previous molecular data for 314 *HER2* unamplified CRCs were available. As there are known differences in the frequencies and distributions of mutations in key oncogenes and tumor suppressors between right and left-sided CRCs, a control group of *HER2* unamplified CRCs was selected after matching tumor sites with 41 *HER2* amplified CRCs. The most common *HER2* amplified tumor site was the left colon (48.8%), followed by the rectum (46.3%) and the right colon (4.9%). A flow chart outlines the selection process of 200 CRCs as a control group after matching tumor sites. The 200 CRCs selected consisted of 5% right-sided colon cancer, 50% left-sided colon cancer, and 45% rectal cancers, with varying frequencies of *KRAS/NRAS/BRAF* mutations.

**Figure 5:**
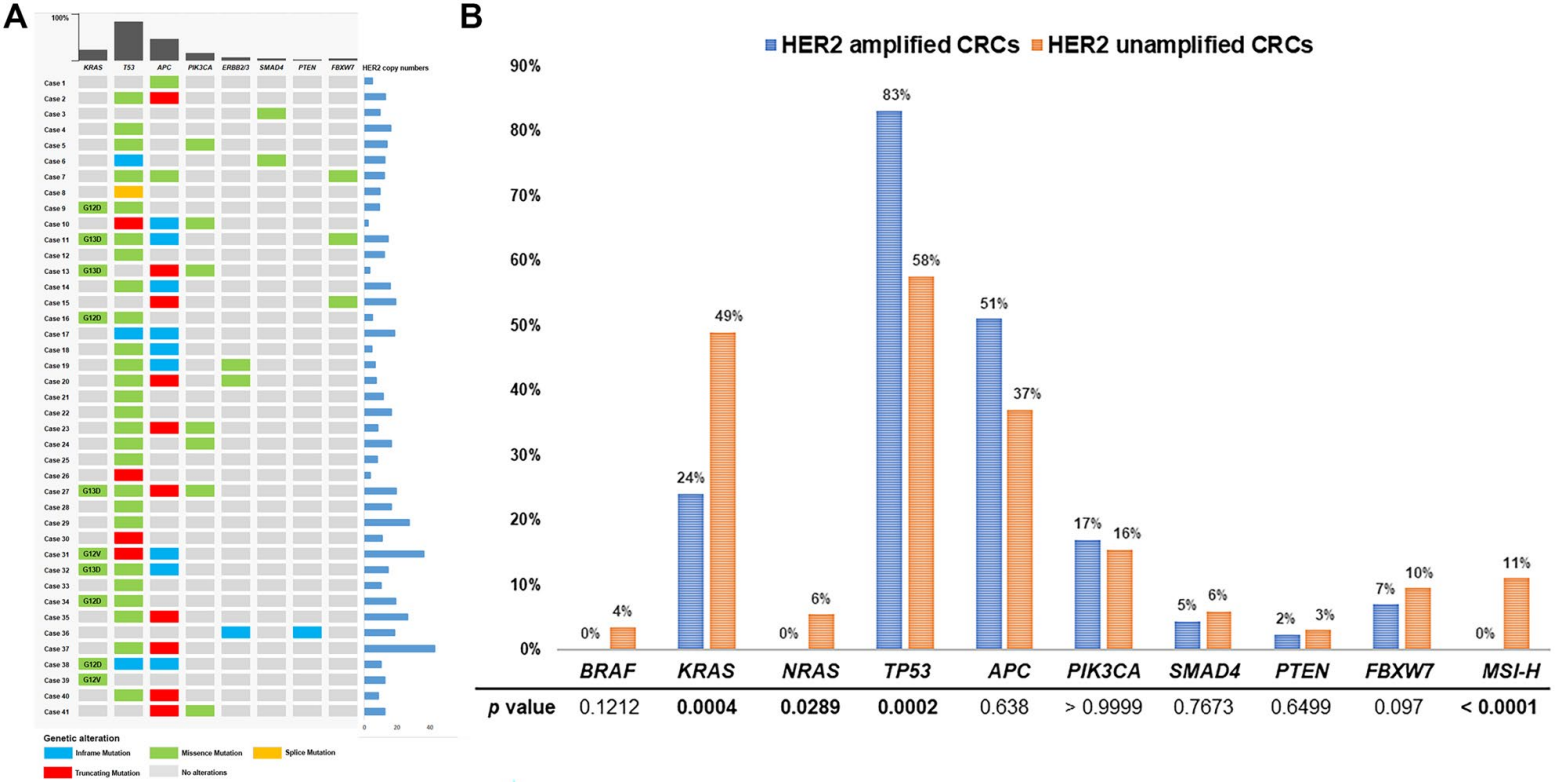
(**A**) Somatic mutational profile and copy number variations of *HER2* in *HER2* amplified CRCs are illustrated. The most frequently identified somatic mutations were found in the TP53 gene, followed by the APC gene. Predominantly, missense mutations were found in *KRAS* and *TP53* mutations. In contrast, mostly truncating and inframe mutations were observed in *APC* mutations. Copy numbers of *HER2* ranged from 5 to 43, with a mean copy number of 13. (**B**) Comparative analysis of frequently identified somatic mutations between *HER2* amplified and unamplified CRCs. No *BRAF* and *NRAS* mutations were identified in *HER2* amplified CRCs. *HER2* amplified CRCs harbored *KRAS* mutations less frequently than unamplified CRCs (24% vs 59%; *p* < 0.0001). In contrast, *TP53* mutations were more frequently identified in *HER2* amplified CRC than unamplified ones (83% vs 62%; *p* = 0.0014). There were no differences in frequencies of other gene mutations. All *HER2* amplified CRCs were MSS phenotypes.

Due to the different prevalences of somatic mutations depending on the anatomical site, molecular data for two hundred CRCs matched by tumor sites were retrospectively collected and analyzed for comparison. A detailed flow chart outlining the selection of 200 CRCs as a control group after matching tumor sites with 42 *HER2* amplified CRCs is illustrated in Fig. 4. Compared with the 200 unamplified CRCs, *TP53* mutations were more commonly observed in *HER2* amplified tumors than unamplified tumors (83% vs 58%; *p* = 0.0002). However, *KRAS* mutations were less frequently identified in the *HER2* amplified tumors than in unamplified tumors (24% vs 49%; *p* = 0.0004). *BRAF *and *NRAS* mutations were not detected in *HER2* amplified tumors but were observed in 4% and 6% unamplified tumors. There were no statistical differences in the frequencies of other genes, including *APC, PIK3CA, SMAD4, ERBB2/3, PTEN, and FBXW7*, which have been reported mutated in CRCs by next-generation sequencing. The somatic mutations and copy numbers of genes identified in the 41 *HER2* amplified CRCs are detailed in Fig. 5A. The results of the comparative analysis of frequently identified mutations in *HER2* amplified and unamplified CRCs after matching tumor sites are depicted in Fig. 5B.

### Clinical outcome

**Figure 6:**
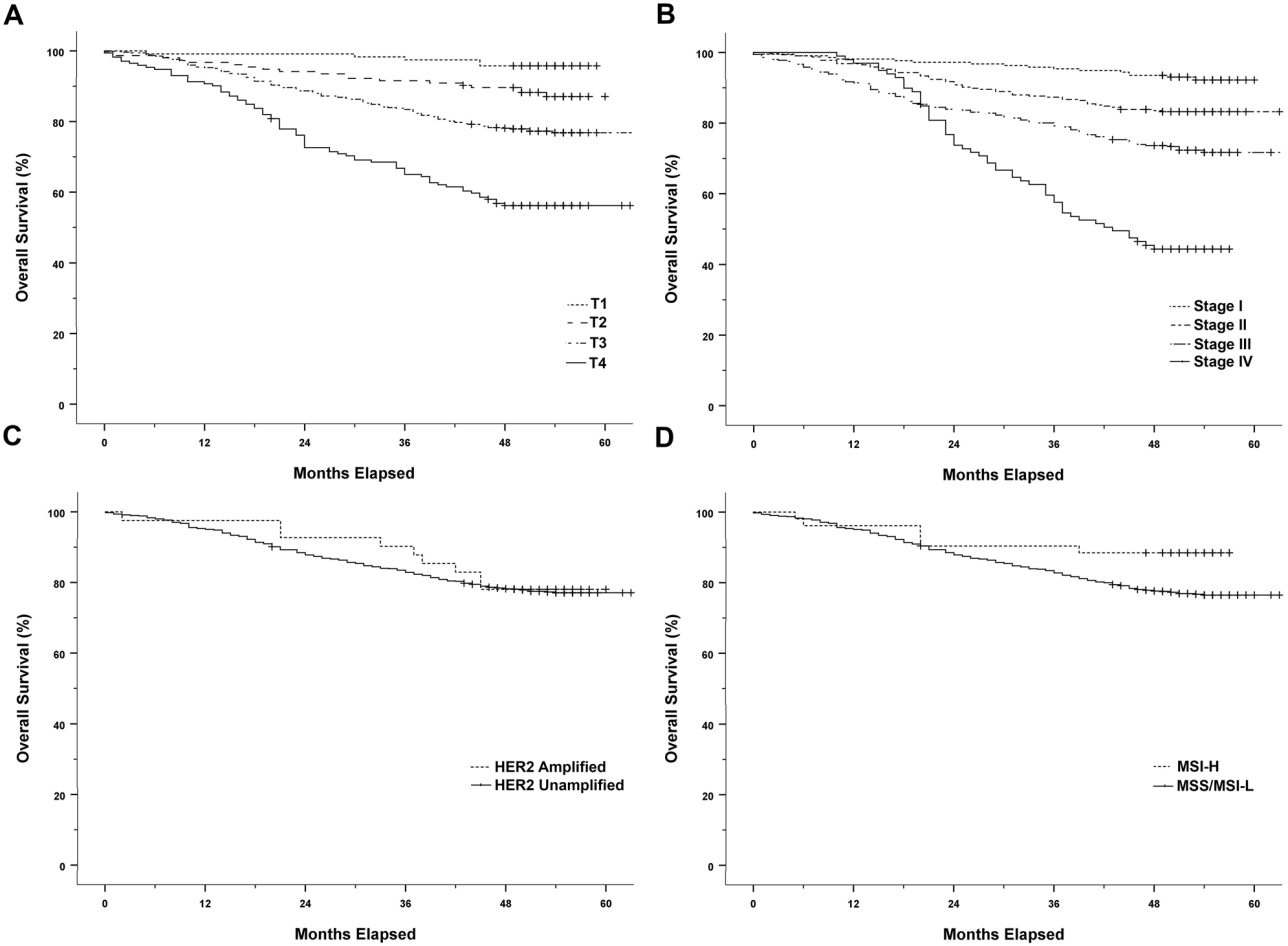
Kaplan–Meier cumulative survival curves. (**A**) Survival according to T category (*p* < 0.001); (**B**) Survival according to pathologic TNM stage (*p* < 0.0001); (**C**) Survival according to *HER2* amplified and unamplified tumors (*p* = 0.827); (**D**) Survival according to MSI-H and MSS/MSI-L tumors (*p* = 0.065).

Follow-up data were available for all 992 patients with primary CRC. The 1-, 3-, and 5-year overall survival (OS) rates for 992 patients were 95%, 83%, and 77%, respectively, with a median follow-up of 52 months (range 1–60 months). Kaplan–Meier survival analysis demonstrated a poor tumor differentiation, high T category, lymphovascular invasion, high N category, distant metastasis at diagnosis (M category), and advanced pathologic TNM stage (all, *p* < 0.0001) to be significantly associated with shorter OS. Regarding potential *HER2* amplification and MSI-H status related to survival, there were no significant differences in OS rates between groups with *HER2* amplified and unamplified tumors (*p* = 0.827) or groups with MSS/MSI-L and MSI-H phenotypes (*p* = 0.065). Kaplan–Meier survival analysis for the T category, pathologic TNM stage, *HER2* amplification, and MSI status are illustrated in Fig. 6.

In the survival analysis of 41 patients with *HER2* amplified CRC, lymphovascular invasion (*p* = 0.015), lymph node metastasis (*p* = 0.018), and pathologic AJCC stage III/IV (*p* = 0.014) were statistically associated with shorter OS. Regarding potential molecular alterations related to survival, there were no differences in OS rates between groups with mutated and wild type *TP53*, *KRAS*, *APC*, *PIK3CA*, and *FBXW7*.

## Discussion

The frequency of *HER2* overexpression/amplification has been reported in 1.3–6.3% of unselected CRCs^1–3^. The Cancer Genome Atlas (TCGA) project detected *HER2* alterations in approximately 7% of patients with CRC, especially in *RAS* and *BRAF* wild type tumors^16^. In this series of 992 patients with primary CRC, forty-one (4.1%) patients were found to have *HER2* amplification. *HER2* amplifications were identified in 1.0% (2/193) of the right colonic tumors, 5.1% (20/394) of the left colonic tumors, and 4.8% (19/400) of the rectal tumors. A retrospective molecular analysis of 1730 CRCs demonstrated that CRC tumors with *HER2* amplification were preferentially located on the left side of the colon^17^. Our study also found that the most common site showing *HER2* amplification was the left colon (48.8%), followed by the rectum (46.3%). Previous and current studies support that *HER2* amplification differs depending on the location of the primary tumor, which tends to be located more frequently in the left colon and rectum. Based on our findings, it would be beneficial to do immunohistochemistry for *HER2* as a screening test on the left-sided CRCs where 95.1% of *HER2* amplifications occur.

There have been conflicting studies on the relationship between *HER2* status and clinicopathologic features in patients with CRC^3,11,13,14^. However, our study demonstrated that there were no significant differences in most clinicopathologic features between amplified and unamplified tumor groups, except for tumor size and MSI status. Moreover, the prognostic significance of *HER2* status in CRCs is disputable. Some studies suggest that *HER2* positivity is related to poor OS, while others have not found any correlation between *HER2* positivity and prognosis^15,18–20^. The present study showed no statistical differences in OS between groups of *HER2* amplified and unamplified CRCs. Although further studies are needed to evaluate the prognostic effect of *HER2* status in CRCs, adding anti-*HER2* inhibitor alone or combination therapy is a promising treatment option for better clinical outcomes in patients with advanced *HER2* amplified CRC.

The rate of MSI-H status in primary CRCs is reported to be 10–15%^6^. The rate of MSI-H phenotype in this study was 10.1% of 992 primary CRCs, which number is within the reported ranges. The relationship between *HER2* amplification and MSI status has yet to be well elucidated. In the MyPathway study, 28 patients with *HER2* amplified metastatic CRCs were MSS^21^. Another molecular study demonstrated that none of the 16 *HER2* amplified CRCs were MSI-H^22^. In this series, all 41 (4.1%) *HER2* amplified CRCs were MSS phenotypes, and all 100 (10.2%) of MSI-H CRCs were unamplified. The prior and current studies suggest that the relationship between *HER2* amplification and MSI-H phenotype in metastatic and primary CRCs is mutually exclusive.

*BRAF* mutation is reported in approximately 5–10% of CRCs^23^. Patients with *BRAF* mutated CRC tend to respond poorly to chemotherapy and progress rapidly with a poorer prognosis compared to those with *BRAF* wild-type CRC^6,24^. Additionally, these patients develop resistance to anti-EGFR inhibitors due to activation of the MAPK pathway downstream of EGFR^25^. Therefore, *BRAF* mutations are considered to be negative predictors of anti-EGFR therapy^26^. Previous studies have also found that 91% of *BRAF* mutations are present in MSI-H CRCs with hMLH1 methylation^27,28^. In this series, 4% of *BRAF* mutations were found in 200 unamplified CRCs, and 90% of *BRAF* mutated tumors were MSI-H. In contrast, no *BRAF* mutations were identified in all 41 *HER2* amplified CRCs, indicating a negative relationship between *HER2* amplification and *BRAF* mutation in CRCs.

The prevalence of *KRAS* mutations is reported to be 30–50% in CRC^29^. *KRAS* mutations are predominantly clustered in exon 2, in codons 12 (near 80% of all *KRAS* mutations), and are less frequent in exons 3 (codons 59 and 61) and 4 (codons 117 and 146) ^30–32^. *NRAS* mutations are uncommon, accounting for 3–5% of CRCs, predominantly in exons 2, 3, and 4^33^. So far, *KRAS* and *NRAS* mutations are associated with clinical resistance to anti-EGFR therapy, which is not recommended for patients with these mutations^10^. However, the incidence of *RAS* mutations in *HER2* amplified CRCs has not been well described. In a study of *HER2* amplification and *KRAS/BRAF* mutation in stage II-III CRC patients, *HER2* amplifications were less frequent in tumors with *KRAS/BRAF* mutations (observed in 0.2–1.4% of tumors harboring *KRAS/BRAF* mutations)^1^. Consistent with this, our study demonstrated that *HER2* amplified CRCs harbored less frequent *KRAS* mutations than unamplified CRCs (24.4% vs 49%; *p* = 0.0004).

*HER2* amplification in CRCs is significantly associated with resistance to anti-EGFR inhibitors and also related to inferior survival outcomes in patients with *HER2* positive tumor exposed to chemotherapy with anti-EGFR inhibitors^34,35^. Therefore, in addition to *RAS/RAF* molecular tests, *HER2* screening is essential for selecting efficacious treatment options for patients with metastatic CRC. According to the updated NCCN guideline, patients with *HER2* amplified and *RAS/RAF* wild type CRC are eligible candidates for fam-trastuzumab deruxtecan-nxki or a combination of trastuzumab with pertuzumab or lapatinib or tucatinib^35^. Our study demonstrated that approximately 75.6% of patients with *HER2* amplified CRC had *RAS/RAF* wild type, suggesting that they may benefit from a combination therapy of anti-*HER2* inhibitors in advanced stages of the disease.

Approximately 60% of CRCs are reported to harbor *TP53* mutation^36^. However, the prevalence rate of *TP53* mutation in CRC varies depending on the anatomical site and tumor molecular subtype^37^. The frequency of *TP53* mutations is higher in distal colon and rectal tumors, while proximal colon tumors with MSI-H and methylator phenotypes have lower frequencies of *TP53* mutations^37,38^.

Frequent *T53* mutations in *HER2* amplified CRCs, up to 83%, were found in this series. In breast cancers, the frequency of *TP53* mutations was reported to be more common in *HER2* positive than *HER2* negative tumors^39,40^. However, in this series of *HER2* amplified CRCs, it is unclear whether frequent *TP53* mutations are associated with *HER2* amplification or are due to a predominant anastomotic site/distal colorectum of *HER2* amplified tumors. Moreover, it is well-known that *TP53* mutations have been associated with a poorer prognosis in CRCs^36,41^. However, we could not find any significant differences in survival between *TP53* mutated versus wild type patients with *HER2* amplified CRC, possibly due to a small number of cases and limited follow-up period. Therefore, further validation studies, including a large-scale series, are needed to investigate *TP53* mutations and associated clinical outcomes in *HER2* amplified CRCs.

The current study has a few limitations. It is a retrospective study conducted at a single institution. The differences in clinicopathologic features and rates of *HER2* amplification may originate from selection biases and interpretation of *HER2* amplification by different methodologies, fluorescence in situ hybridization versus. DISH. However, the concordance rate of *HER2* amplification between results of FISH and next-generation sequencing was 100% in this study. In addition, molecular data for a control group was retrospectively collected from patients who underwent next-generation sequencing with different numbers of gene panels (80–559 gene panels). Therefore, only frequently identified somatic mutations in CRCs between the study and control groups were compared.

In summary, *HER2* amplifications were found in 41 (4.1%) of 992 primary CRCs. All 41 *HER2* amplified tumors were MSS. In addition, *HER2* amplified CRCs showed a lower rate of *KRAS* mutations (24.4%) but a higher rate of *TP53* mutations (83%) than unamplified CRCs. No *BRAF* and *NRAS* mutations were identified. Our study suggests that *HER2* amplified CRCs are mutually exclusive of microsatellite instability and harbor less frequent *KRAS/NRAS/BRAF* mutations but frequent *TP53* mutations.

## Methods

### Patient selection and clinicopathologic evaluation

We reviewed pathology records of patients diagnosed with primary colorectal adenocarcinoma from the Pathology Department at JNUH. Thousand twelve surgically resected cases were initially searched and reviewed from 2017 to 2022. Among them, cases with colorectal adenocarcinoma in situ (Tis) were excluded. A total of nine hundred ninety-two surgical cases of invasive CRCs were selected. Histologic findings were re-evaluated, including tumor size, tumor differentiation, depth of invasion, lymphovascular invasion, perineural invasion, regional lymph node metastasis, distant metastasis, and pathologic TNM staging. Tumors were restaged according to the 8th edition of the American Joint Committee on Cancer (AJCC) TNM staging system for CRCs after the clinicopathologic features and radiologic findings had been re-reviewed^42^. Demographic and clinical information, including sex, age, type of surgery, tumor site, date of diagnosis, preoperative neoadjuvant therapy, and last follow-up status, were collected from reviews of the patient’s medical records. This study was approved by the institutional review board (IRB) of Jeju National University Hospital (IRB number: JEJUNUH 2022-01-013), with written patient consent deemed unnecessary.

### Immunohistochemistry and DISH for *HER2*

Immunohistochemistry was performed on a representative tissue section of each case using the avidin–biotin method. The primary antibody was against *HER2*/neu (rabbit monoclonal antibody clone 4B5, prediluted, Ventana Roche, USA). An automated stainer (Ventana Medical Systems, AZ, USA) was used per the manufacturer’s protocol. The level of *HER2* expression was evaluated according to Hofmann’s scoring system used for surgical specimens as follows: 0, no reactivity or membranous activity in < 10% of tumor cells; 1 + , faint or barely detectable membranous reactivity in ≥ 10% of tumor cells; 2 + , weak to moderate complete, basolateral or lateral membranous reactivity in ≥ 10% of tumor cells; and 3 + , moderate to strong complete or basolateral membranous reactivity in ≥ 10% of tumor cells^43^.

After reviewing the immunohistochemical results of *HER2* proteins, DISH was performed with cases with IHC scores of 1 + , 2 + , and 3 + . DISH was performed on a Bench Mark XT (Ventana Medical Systems) using the FDA-approved INFORM *HER2* DISH DNA Probe Cocktail Assay (Ventana Medical Systems), which contains a digoxigenin (DIG)-labeled probe for the ERBB2 locus and a dinitrophenyl (DNP)-labeled probe for the centromeric region of chromosome 17. Briefly, 4-μm sections from each case were cut and deparaffinized. They were incubated in Cell Conditioning 2 (CC2) solution followed by protease digestion with ISH Protease 3. After applying 10 μl of INFORM *HER2* Dual ISH DNA probe, the genomic DNA in tissue sections and the nick-translated *HER2* probes were codenatured by heat treatment and hybridization. *HER2* signals were detected using an UltraView DISH DNP and Red ISH DIG Detection Kit. The *HER2*/*CEP17* ratio was determined by counting the *HER2* and *CEP17* signals in 20 nuclei for each section. *HER2* gene amplification was defined as a DISH result showing a *HER2*/*CEP17* ratio of ≥ 2.0^44^. When the *HER2*/*CEP17* ratio was between 1.8 and 2.2, twenty additional cells were recounted in an alternative area and added.

### Microsatellite instability testing

Microsatellite instability (MSI) analysis was performed on all colorectal tumors by multiplex polymerase chain reaction (PCR) with five quasi-monomorphic mononucleotide repeat markers: BAT25, BAT26, D5S346, D17S250, and D2S123. Genomic DNA was isolated from paraffin-embedded tumor tissues using a QIAamp DNA Mini Kit (Qiagen, CA, USA). Each primer was end-labeled with one of the following fluorescent markers: FAM, HEX, or NED. An ABI Prism 3130 Genetic Analyzer (Applied Biosystems, CA, USA) was used to analyze the products, and allelic sizes were estimated by Genemapper 4.1 (Applied Biosystems, CA, USA). Tumors with allelic size variation in two or more microsatellite markers were deemed microsatellite instability-high (MSI-H), whereas tumors with allelic variations in one of the microsatellites were classified as microsatellite instability-low (MSI-L). If there were no allelic size variations, all microsatellites were considered microsatellite-stable (MSS).

### Targeted next-generation sequencing

Deep targeted DNA sequencing was performed on all 41 *HER2* amplified tumor samples. After a review of matched hematoxylin and eosin-stained slides, formalin-fixed and paraffin-embedded (FFPE) tissue blocks containing adequate tumor cellularity (> 70%) were selected by S.L. Genomic DNA was extracted using QIAamp DNA FFPE Tissue Kit (Qiagen, Valencia, CA, USA), and quantified using a Qubit dsDNA HS Assay Kit with the Qubit 1.0 fluorometer (Life Technologies, Carlsbad, CA, USA). A NanoDrop spectrophotometer (Thermo Fisher Scientific, Waltham, MA, USA) was used to measure the concentration, and an initial quality check was performed using electrophoresis on 1% agarose gel, loading 0.5 µl of DNA solution. Deep targeted DNA sequencing was performed using the NextSeq 500 system (Illumina, San Diego, CA, USA) with an 88-gene panel mainly focusing on gastrointestinal cancers. Briefly, the cancer-related genes evaluated included ABL1, *AKT1, AKT3, ALK, APC, BRAF, BRCA1, BRCA2, CDH1, CDKN2A, EGFR, ERBB2, ERBB3, FBXW7, GNAS, HRAS, IDH1, IDH2, KRAS, MLH1, NOTCH1, NRAS, PDGFRA, PIK3CA, PTEN, RB1, SMAD4, SMARCB1, TP53, VHL, WT1* among others. Using the hybrid capture method, DNA libraries were prepared using an Agilent SureSelect^XT^ Target Enrichment Kit (Agilent Technologies, Santa Clara, CA, USA). DNA libraries that passed quality checks were sequenced using NextSeq 500. Library preparation and deep targeted sequencing were performed at Macrogen Incorporate (Seoul, Republic of Korea). Library preparation and targeted sequenced reads were mapped to the human reference genome hg38 using the Burrows-Wheeler Aligner-MEM algorithm with default options^45^. Variants were called by the Genome Analysis Tool Kit (GATK) v4.0.9.1^46^ and annotated using SnpEff & SnpSift v4.3i^47^ and dbNSFP v2.9.3^48^. False-positive variants were manually curated using Integrative Genomic Viewer^49^. Amplification of *HER2* was defined as ≥ 5 copies of *HER2* above the average ploidy of the tumor sample^50^. Among 951 unamplified CRCs, we retrospectively identified and collected results of next-generation sequencings with different gene panels (80–559 gene panels) of 200 primary CRCs after matching tumor sites as a control group to compare frequently identified somatic mutations with *HER2* amplified CRCs.

### Statistical analysis

Pearson’s chi-square test or Fisher’s exact test was applied to evaluate correlations between clinicopathologic variables and frequently mutated genes. Continuous variables were analysed using Student’s *t*-test or the Mann–Whitney *U*-test. Cumulative survival rates were calculated by the Kaplan–Meier method. Significant differences in survival status were evaluated using log-rank and Wilcoxon tests. The statistical analyses were performed using SPSS 24.0 software (SPSS Inc., Chicago, IL). A *p*-value < 0.05 was considered statistically significant.

### Ethical approval and consent to participate

The study was conducted in accordance with the guidelines of the Declaration of Helsinki and approved by the Institutional Review Board of Jeju National University Hospital (IRB File No. 2022-01-013). The informed written consent was also obtained from the subjects involved in this study.

### Supplementary Information

Supplementary Table 1.

## Data Availability

Data is provided within the manuscript and supplementary information files.
