# Supplementary material for: RAS/RAF mutations and microsatellite instability status in primary colorectal cancers according to HER2 amplification
Source: Sci Rep. 2024 May 19;14:11432. doi: 10.1038/s41598-024-62096-x (PMC11102903; doi:10.1038/s41598-024-62096-x)
Supplement: Supplementary file 1 — Supplementary Table 1. [file 41598_2024_62096_MOESM1_ESM.docx]

Supplement Table 1 Somatic mutations and gene alterations in 41 HER2 amplified CRCs

| **Gene** | **Somatic mutations and gene alterations** | **Number** |
| --- | --- | --- |
| *KRAS* | G12D, G12V, G13D | 10 |
| *TP53* | C242fs*5, E286K, E285Gfs*60, G245S, G302fs*4, K132R,  M133K, P191T*, Q192*, R110L, R175H, R196P, R213*,  R248Q, R248W, R273H, R282W, V272L, V272M, Y126C,  Y234C, 919+1G>A | 34 |
| *APC* | A462G, E941*, E1306*, E1317Q, E1322*, E1345*, H1349fs,  H1349fs*19, M1431fs*42, P1442fs*31, Q1367*, Q1378*, Q1406*,  Q1429*, Q1447fs, R1450*, R1463Efs*10, R1920Q, S1117F, S1315*, S1356*, S1501fs*7, T872fs*42, T1293fs*14, X176_splice, X516_splice | 21 |
| *PIK3CA* | E545Q, L1067F, M1043I, N107S, Q546H, Q546K, R88Q, T1025A | 7 |
| *ERBB2/3* | V104L, V777L | 3 |
| *SMAD4* | A118V, I179V | 2 |
| *PTEN* | K144Nfs*3 | 1 |
| *FBXW7* | R479Q, R505C | 3 |

* stop codon; fs, frameshift mutation
